# F6H8 as an Intraoperative Tool and F6H8/Silicone Oil as a Postoperative Tamponade in Inferior Retinal Detachment with Inferior PVR

**DOI:** 10.1155/2014/956831

**Published:** 2014-01-02

**Authors:** Gian Marco Tosi, Davide Marigliani, Tommaso Bacci, Napoleone Romeo, Angelo Balestrazzi, Gianluca Martone, Tomaso Caporossi

**Affiliations:** ^1^Department of Medicine, Surgery and Neuroscience, University of Siena, Siena, Italy; ^2^Section of Ophthalmology, Department of Medicine, Surgery and Neuroscience, University of Siena, 53100 Siena, Italy; ^3^Department of Ophthalmology, Catholic University Sacro Cuore-Policlinico A. Gemelli, Rome, Italy

## Abstract

*Purpose*. To evaluate the effectiveness and safety of perfluorohexyloctane (F6H8) for intraoperative flattening of the retina and of F6H8/silicone oil (SO) 1000 cSt as a postoperative tamponade for inferior retinal detachment with inferior proliferative vitreoretinopathy. *Methods*. This is a retrospective review of 22 patients who underwent pars plana vitrectomy using F6H8 as an intraoperative tool to flatten the retina. At the end of the surgery a direct partial exchange between F6H8 and SO 1000 cSt was performed, tamponing the eye with different ratios of F6H8/SO (70/30, 60/40, 50/50, 40/30, and 30/70). Anatomical and functional results and complications were evaluated over the follow-up period (mean 22.63 months). *Results*. F6H8 was efficacious for intraoperative flattening of the retina. Twenty-one of the 22 patients achieved a complete retinal reattachment. Postoperative visual acuity (VA) ranged from light perception to 20/70, with 72% of patients obtaining VA better than 20/400. No emulsification/inflammation was observed whatever the ratio of F6H8/SO used. With higher ratios of F6H8/SO (70/30 and 60/40) cloudiness of the tamponade was observed. A transparent mixture was present with all the other ratios. *Conclusions*. The surgical technique adopted is very simple and safe. The optimal F6H8/SO ratio seems to be between 50/50 and 30/70.

## 1. Introduction

The complexities associated with inferior retinal detachment (RD) complicated by inferior proliferative vitreoretinopathy (PVR) are well recognized [1]. In attempts to reduce the postoperative burden for patients, vitreoretinal surgeons have tested various substances with a specific gravity higher than water [1]. Fluorinated SO and perfluorocarbon liquids (PFCLs) have been used as alternative internal tamponades but are not well tolerated as long-term internal tamponades [1–4]. In the early 2000s partially fluorinated alkanes (FALKs) were introduced as long-term heavy tamponades [1]. Perfluorohexyloctane (F6H8) has a density of 1.3 g/cm^3^. Although its tolerability and biocompatibility have been demonstrated in the experimental animal and in human eyes, early F6H8 dispersion and emulsification with consequent inflammatory responses have frequently been reported [5–8]. The use of SO in combination with FALKs (heavy silicone oil, HSO), thereby increasing the viscosity of FALKs, has been suggested to reduce emulsification [9, 10]. Four different prefabricated mixtures of FALKs and SO of varying specific gravities and viscosities are now available: Oxane HD, Densiron 68, HWS 46–3000, and HWS 45–3000. All these mixtures include high viscosity SO, ranging from 5000 to 100000 cSt [1, 11–17]. While encouraging results have been published, difficulties associated with the intraoperative handling of the substance (e.g., air-heavy tamponade exchange, heavy liquid-heavy tamponade exchange, or heavy tamponade removal), as well as postoperative side effects, have also been reported [18–21]. Before prefabricated mixtures were available, dual filling with F6H8 and SO 1000 cSt was carried out in two selected series of patients with inferior PVR by Tognetto et al. [22] and Rizzo et al. [23], with excellent results. Tognetto et al. [22] injected a mixture of 3 mL F6H8 and 7 mL of 1000 cSt SO, while Rizzo et al. [23] performed a sequential injection of 70% F6H8 and 30% SO 1000 cSt. When either prefabricated mixture or dual fillings were used, almost all vitreoretinal surgeons chose to inject the heavy tamponade after heavy liquid-air exchange, obtaining intraoperative flattening of the retina with the heavy liquid [1]. In the present series we, retrospectively, analyze the outcomes of patients with inferior RD complicated by inferior PVR, who were operated on using F6H8 as an intraoperative tool to flatten the retina and in whom a direct partial exchange between F6H8 and SO 1000 cSt was performed. The eyes were tamponed with different quantitative ratios of F6H8 and SO (70/30, 60/40, 50/50, 40/30, and 30/70). Our experience with a relatively long followup is presented.

## 2. Materials and Methods

We performed a retrospective evaluation of 22 patients with inferior retinal detachment and inferior retinal breaks complicated by severe inferior PVR, who were operated on between 2007 and 2011 by means of pars plana vitrectomy (PPV). F6H8 was used as an intraoperative tool to flatten the retina, and at the end of the surgery F6H8 was partially replaced with SO 1000 cSt. This procedure was chosen because all the patients of the series declared to be unable to maintain an appropriate postoperative face-down positioning which would have been required with conventional tamponade. In all eyes PVR had a grade greater than CP-6 and greater than CA-6 (updated PVR classification) [24].

Perfluorohexyloctane (Fluoron, Germany) has the following physical properties: density of 1.3 g/cm^3^, viscosity of 2.5 mPa · s, and an interface tension against water of 49.1 mN/m.

Initial clinical examination included a detailed history of ophthalmic surgery, a complete ophthalmologic examination with measurement of best-corrected visual acuity (VA) using the Early Treatment Diabetic Retinopathy Study tables, measurement of intraocular pressure (IOP), and detailed fundus examination. The RD was described, noting the grade of PVR, using the updated classification of the Retina Society [24]. The same examinations were carried out at all follow-up visits throughout the study.

Our patients comprised 10 women and 12 men, whose ages ranged from 34 to 84 years (mean: 69 years). Three eyes were phakic, 1 was aphakic, and 18 were pseudophakic. Five patients had high myopia (more than six diopters). The patients' preoperative characteristics are given in Table 1.

Ten patients were not operated on for retinal detachment before undergoing PPV surgery with F6H8. Of the 12 patients who had previous vitreoretinal surgery, 8 (nos. 4, 5, 6, 8, 10, 12, 14, and 22) underwent PPV with scleral buckle (SB), 2 (nos. 3 and 9) underwent PPV without SB, and 2 (nos. 2 and 7) underwent SB without PPV.

All patients underwent PPV using the Millennium vitrectomy system (Bausch & Lomb Inc., St. Louis, MO). A scleral buckling procedure was performed using a silicone encircling band in 11 (nos. 1, 3, 11, 13, 15, 16, 17, 18, 19, 20, and 21) of the 12 patients without prior buckling surgery and was associated with a radial element in 1 patient (no. 21) who had an inferior posterior break. In 1 patient (no. 9) placement of an encircling band was not possible due to extensive adherences under the lateral rectus muscle as a consequence of previous glaucoma surgery (trabeculectomy with mitomycin). In this case a 90° segmental buckle was placed in the inferonasal quadrant. Ten patients (nos. 2, 4, 5, 6, 7, 8, 10, 12, 14, and 22) underwent a scleral buckling procedure before PPV with F6H8. In these cases, we decided not to revise the SB to save time (much work had to be done on the vitreous, epiretinal, and subretinal membranes). In the 3 phakic patients (nos. 1, 17, and 21) the lens was removed by performing phacoemulsification through a clear corneal tunnel at the time of vitrectomy. This was followed by implantation of an intraocular lens (IOL) in 1 patient (no. 1), while 2 patients (no. 17 and 21) were left aphakic (although the capsular support was intact) to enhance visualization during PPV.

In all patients, the vitreous base was shaved using the vitrector, and all epiretinal membranes were removed. Subretinal membranes were removed when deemed necessary to achieve retinal flattening. In 3 patients (nos. 3, 4, and 7) it was not possible to achieve inferior retinal mobility due to severe contraction, so a peripheral retinectomy was performed. In another patient (no. 17) inferior retinectomy was associated with the removal of a wooden epiretinal foreign body.

Once proper retinal mobility was achieved, the retina was flattened with pure F6H8. Endophotocoagulation was subsequently performed around the breaks and retinectomy edges using a diode laser. The operation was concluded with a direct partial exchange between F6H8 and SO 1000 cSt. In particular, when the light fiber had been inserted into the 2 o'clock sclerotomy and a passive unprotected Charles flute cannula was inserted into the 10 o'clock sclerotomy, SO 1000 cSt was actively injected through the infusion line. The exchange was stopped at 70% of the vitreous cavity in 3 patients (nos. 1, 2, and 3), at 60% in 7 patients (nos. 3, 4, 5, 6, 7, 8, and 9), at 50% in 3 other patients (nos. 9, 10, and 11), at 40% in 2 patients (nos. 12 and 13), and at 30% in 9 patients (nos. 14, 15, 16, 17, 18, 19, 20, 21, and 22) (patients 3 and 9 were operated on twice). To obtain the desired quantitative ratio of F6H8 to SO, the volume of the vitreous cavity was calculated in cubic millimeters by measuring the length of the vitreous cavity, using standardized A scan ultrasonography, and multiplying it by a coefficient of 315 [23]. The average error was 8–10%. In the eyes filled with SO, the vitreous cavity length was measured using a sound speed of 980 m/s for SO 1000 cSt or 1040 m/s for SO 5000 cSt.

The ratio of F6H8 to SO was changed over time according to our observations during the postoperative period in the first patients of the series (see Section 3).

In 21 patients the tamponade was removed via the pars plana between the third and the fifth month after surgery. In 1 patient (no. 16) the tamponade has not yet been removed due to her compromised general health conditions, which have not allowed her to undergo surgery.

The follow-up period was between 12 and 48 months (mean 22.63 months).

We observed the efficacy and safety of F6H8 as an intraoperative tool for retinal flattening during PPV, the best ratio of F6H8 to SO in the postoperative period, the duration of presence of the F6H8/SO tamponade, the technical difficulties encountered in F6H8/SO removal, and the final anatomical and functional outcome, including any complications.

## 3. Results

F6H8 was efficacious as an intraoperative agent used to flatten the retina in all cases. The interface of the F6H8 bubble inside the eye was not as visible as that of conventional perfluorocarbon liquid.

In the first 9 patients (nos. 1, 2, 3, 4, 5, 6, 7, 8, and 9) of the series the exchange was stopped when there was still more F6H8 than SO inside the eye, to be sure of an effective inferior retinal tamponade postoperatively (ratios of 70/30, 60/40). In these cases at fundus examination on postoperative day 1, with the patient in a seated position, we observed two phases (F6H8 and SO, not mixed together), with an interface above the superior arcades when the ratio was 70/30 and at the level of the superior arcades when the ratio was 60/40. In all of these 9 patients (3 (nos. 1, 2, and 3) with a ratio of 70/30 and 7 (nos. 3, 4, 5, 6, 7, 8, and 9) with 60/40 (patient no. 3 was operated on twice with F6H8)) few days after surgery and throughout the entire follow-up period with the tamponade in the eye, at the beginning of fundus visualization, the two substances appeared amalgamated with a clear fundus visualization but after a few seconds we observed a cloudiness of the tamponade in the posterior pole and in the inferior quadrants. This two-phase phenomenon prevented precise examination of the fundus. Based on the above mentioned results, we lowered the ratio of F6H8 to SO to 50/50 in 3 patients, to 40/60 in 2 patients, and to 30/70 in 9 patients. In these cases (ratio: 50/50 to 30/70) on postoperative day 1 there was a visible interface just below the optic nerve (ratio: 50/50) (patients nos. 9, 10, and 11), below the inferior arcade (ratio: 40/60) (patients nos. 12 and 13), or further down (ratio: 30/70) (patients nos. 14, 17, 19, 20, and 21). In the other 4 patients (nos. 15, 16, 18, and 22) with a 30/70 ratio no interface was visible and the two substances were perfectly amalgamated on the first day after surgery. In the course of followup with the tamponade inside, just a few days after surgery, only one phase was observed in the eyes of patients with ratios of 50/50, 40/60, and 30/70 who had shown two phases on postoperative day 1. No further changes were observed in these patients during the rest of the follow-up period. In all these patients, including those with a 30/70 ratio who showed a single phase from postoperative day 1, the phase remained single throughout followup and no cloudiness was observed during fundus examination, allowing the physician to follow up the eye very carefully and perform laser if necessary.

During the followup 1 patient (no. 1) with a F6H8/SO ratio of 70/30 showed increased IOP the day after surgery, which was controlled with medications for the next 20 days, until it returned to be within normal limits. Five patients, with ratios of 60/40 (3 (nos. 6, 8, and 9)), 50/50 (2 (nos. 9 and 10)), and 30/70 (1 (no. 17)) (patient no. 9 was operated on twice with F6H8), showed low IOP, which remained in the low range throughout the follow-up period, without any clinical signs of hypotony. During the follow-up period posterior synechiae were observed in 5 patients: 1 (no. 1) with a F6H8/SO ratio of 70/30, 1 (no. 8) with 60/40, 1 (no. 9) with 60/40 in the first operation and 50/50 in the second operation, and 2 (nos. 16 and 21) with 30/70. In 1 patient (no. 13) inferior retinal hemorrhages were observed but were resolved one month after surgery (40/60 ratio). Two patients (nos. 18 and 19), both with a ratio of 30/70, developed cystoid macular edema, as determined by OCT examination.

Finally, in 1 patient (no. 11) with 50/50 ratio a migration of the tamponade into the anterior chamber occurred through a partial inferior zonular dehiscence, forming bubbles of the F6H8/SO mixture in the anterior chamber but not causing corneal damage.

No patient showed emulsification or signs of vasculitis or uveitis.

Regarding retinal behavior during the presence of the F6H8/SO tamponade, we observed retinal attachment without progression of PVR in 16 out of 22 patients (72%). Among the remaining patients, we observed recurrent inferior PVR in 2 (nos. 3 and 9) (ratios of 70/30 and 60/40, resp.) and recurrent tractional RD with superior PVR in 3 (nos. 14, 17, and 22) (all with 30/70 ratio). Due to her compromised general conditions 1 patient (no. 16) was followed up for 14 months without F6H8/SO removal: no signs of inflammation or uveitis were observed, except for the formation of mild posterior synechiae; the retina remained attached although a pucker was observed.

Except for the last patient mentioned, all the other patients underwent tamponade removal. The F6H8/SO mixture was removed very easily via the pars plana, by means of manual active aspiration through a cannula, three-to-five months after surgery. The resistance of the tamponade to aspiration was very low. At the end of the active aspiration of the mixture we left a small bubble of F6H8/SO on the surface of the posterior retina, which was removed with an unprotected Charles flute cannula. No adhesion of the mixture to the surface of the retina was observed and no dispersion was noted in either the vitreous cavity or the anterior chamber. During tamponade removal in all the patients with 70/30 and 60/40 ratios the F6H8/SO was very cloudy, while in those with lower ratios it remained transparent. In 1 patient (no. 7) removal of the F6H8/SO mixture was associated with residual membrane peeling and gas tamponade. In 2 patients (nos. 6 and 8) with low IOP, removal of F6H8/SO was followed in the same procedure by SO 1000 cSt tamponade. In these 2 patients SO was not removed; no signs of emulsification were observed after 28 and 36 months, respectively, and IOP remains in the low range without clinical signs of hypotony.

One patient (no. 2) with high myopia, who presented with recurrent inferior RD with inferior PVR after two SB procedures, underwent PPV with a F6H8/SO tamponade (70/30 ratio). In the followup the retina remained flat and the mixture was removed three months after PPV. After removal the retina remained flat for the first six months. The patient was not seen for followup in the subsequent six months; then, 12 months after removal, he returned to us showing a significant inflammatory reaction of the eye and recurrent shallow RD with retinal ischemia. He denied further surgery.

The 2 patients (nos. 3 and 9) with recurrent inferior PVR underwent F6H8/SO removal and PPV with PVR removal and were resubjected to tamponade with F6H8/SO after flattening the retina intraoperatively with pure F6H8 (as in the first operation with F6H8). At the end of the operation a direct partial exchange with SO cSt was performed, obtaining F6H8/SO ratios of 60/40 and 50/50, respectively. In these 2 patients, the behavior of F6H8/SO in the postoperative period was the same as described in the first procedure in terms of temporary cloudiness. No vasculitis, uveitis, active inflammation, or emulsification was observed. The mixture was efficacious in flattening the retina in both patients and no recurrence of PVR was observed in the follow-up period. One (no. 9) of these 2 patients with low IOP after the first operation with F6H8/SO showed low IOP after the second operation with F6H8/SO, but without clinical signs of hypotony. The tamponade was removed after three months in 1 patient (no. 3) and after four months in the other (no. 9); at the end of the surgery the eye with normal preoperative IOP (no. 3) was left without a tamponade, while the eye with low preoperative IOP (no. 9) was left with gas. The patient with low preoperative IOP returned to normal IOP levels after four months.

The 3 patients (nos. 14, 17, and 22) with recurrent RD and superior PVR underwent F6H8/SO removal, PPV, PVR removal, and intraoperative flattening of the retina with conventional perfluorocarbon liquid and direct complete exchange between perfluorocarbon liquid and SO 1000 cSt. All of these 3 patients showed a flat retina in the followup without recurrence of PVR. Two of them (nos. 14 and 22) with normal IOP underwent SO removal, while the other (no. 17) did not undergo SO removal due to low IOP. In the latter patient SO is still in the eye after 12 months, without signs of emulsification and IOP remains in the low range without signs of hypotony.

In summary, at the end of the follow-up period the retina was attached in 21 out of 22 patients (17 without a tamponade, 3 with a SO 1000 cSt tamponade, and 1 with a F6H8/SO tamponade). The only patient with retinal detachment was no. 2.

The functional results are presented in Table 1. VA values ranged from LP to 20/70. Sixteen patients (72%) had VA better than 20/400. Before entering the study 20 patients (90%) had VA below 20/400 and 2 patients (9%) had VA of 20/400. Comparing preoperative to postoperative data, VA was unchanged in 2 patients (9%), worse in 1 patient (4.5%), and better in 19 patients (86%).

## 4. Discussion

Four prefabricated mixtures of FALKs and SO, differing in specific gravities and viscosities, are now available: Oxane HD, Densiron 68, HWS 46–3000, and HWS 45–3000 [1, 11–17]. All mixtures are composed of high viscosity SO, ranging from 5000 to 100000 cSt [1, 11–17]. Encouraging results have been shown using all of them, although many authors have observed postoperative complications including dispersion or emulsification and intraocular inflammation [20, 21]. Moreover, difficulties associated with the intraoperative handling of the substances have been reported. In fact, most vitreoretinal surgeons inject heavy tamponade after heavy liquid/air exchange, thus running the risk of slippage of the retinotomy edge in the case of large retinotomies [1]. In order to avoid retinotomy slippage, a direct heavy liquid/heavy tamponade exchange can be performed; however, this procedure may lead to a “contamination” of the heavy tamponade, thus increasing the risk of emulsification, “sticky” SO, and inflammatory response [1]. Due to the high viscosity, difficulties in heavy tamponade removal have also been reported, including the need to enlarge the sclerotomy, risk of posterior retinal damage, and collapse of the eye. This has stimulated vitreoretinal surgeons to seek alternative and safer methods for heavy tamponade removal [25–27].

Before prefabricated mixtures became available, dual filling with F6H8 and SO 1000 cSt was used in two selected series of patients with inferior PVR, by Tognetto et al. [22] and Rizzo et al. [23]. Tognetto et al. [22] injected a mixture prepared in a syringe of 3 mL F6H8 and 7 mL polydimethylsiloxane 1000 (the syringe was shaken for a few seconds), while Rizzo et al. [23] used a sequential injection of 70% F6H8 and 30% SO 1000 cSt. Both Authors reported excellent anatomical results. Moreover, Rizzo et al. [28] reported the outcome of another series of patients with severe RD, including cases with inferior RD and inferior PVR, who were tamponed with a dual filling of F6H8 (70%) and SO 1000 cSt (30%) and showed good intraocular tolerance of the mixture. Both Tognetto et al. [22] and Rizzo et al. [23, 28] chose to inject the heavy tamponade after heavy liquid-air exchange, obtaining intraoperative flattening of the retina with heavy liquid.

F6H8 was initially used as an intraoperative agent to flatten the retina and was left in the eye without SO as a long-term heavy tamponade in several clinical trials. Although F6H8 has been reported as being efficacious and without side effects in intraoperative flattening of the retina, its postoperative use not combined with SO has been discontinued due to the postoperative development of intraocular inflammation and emulsification in an elevated percentage of cases [5–8].

In the present series, as also reported by Kirchhof et al. [5] and by Roider et al. [6], F6H8 was easy to inject and efficacious in all cases as an intraoperative agent to flatten the retina. Although we could not perform a direct comparison with conventional perfluorocarbon liquid, as F6H8 has a lower specific gravity than conventional perfluorocarbon liquid, it seems to require a more few minutes to obtain an appropriate flattening of the retina. We suggest that injecting the intraoperative tamponade only after PVR removal has been completed and/or retinotomy has been performed, to avoid the risk of tamponade migration under the retina. We did not experience any F6H8 migration under the retina and succeeded in flattening the retina in every patient. The interface of the F6H8 bubble inside the eye is not as visible as that of conventional perfluorocarbon liquid and consequently the surgeon has to exercise caution, especially during the exchange with SO. In all our patients the F6H8/SO mixture was removed very easily via the pars plana, between the third and the fifth month after surgery. The resistance of the tamponade to aspiration was very mild, probably due to the low viscosity of the mixture.

Our results are in agreement with those of Tognetto et al. [10], who showed that a 30/70 ratio is the optimal ratio to obtain an optically clear single-bubble mixture and maintain a good tamponade effect on the inferior retina. In our series 4 of the 9 eyes with a F6H8/SO ratio of 30/70 showed a single bubble from day 1 after surgery (no visible interface), no cloudiness during fundus examination, and good inferior retinal tamponing. In almost all cases of their series with a ratio of 30/70 Tognetto et al. [22] noted a superior meniscus between the heavy oil bubble and water. None of the patients in our series with a 30/70 ratio showed a superior meniscus; they showed excellent fundus visualization even in the extreme superior periphery of the retina and good inferior retinal tamponing.

Safety and efficacy of the mixture were also observed in the eyes tamponed with higher ratios of F6H8/SO: no inflammation/emulsification or negative effects on postoperative retinal flattening were observed. The only drawback with higher ratios of F6H8/SO was hampered retinal visualization during followup. In particular, with ratios of 70/30 and 60/40 we experienced a clouding effect after a few seconds of fundus visualization, making the followup challenging but not impossible. The cloudiness with these quantitative ratios might be explained by micelle formation of F6H8 as a function of surfactant concentration and is possibly related to eye movements or to the patients' exposure to cold [1, 29]; in our series this phenomenon seemed to be induced by light during ophthalmoscopy, perhaps due to its heating effect. In our opinion this two-phase phenomenon, which may occur under different circumstances, is transitory, thus preventing PVR formation at the interphase.

With the 50/50 and 40/60 ratios the surgeon has to wait for between two to three days and a week to visualize a single mixture during fundus examination, although visualization is good even when two phases are present.

The preoperative method of vitreous volume measurement adopted in the present series to obtain the desired F6H8/SO ratio is less precise if compared to the use of prefabricated mixtures with known F6H8/SO ratios. Although the amount of F6H8 injected intraoperatively to fill the vitreous cavity in order to obtain retinal flattening confirmed our preoperative calculations, we believe that a small percentage of error still exists. However, this small percentage of error (8–10% maximum) is unlikely to have a jeopardizing effect on the final outcome.

In our series, PVR recurrence during followup with a tamponade in the eye was observed with different quantitative ratios of F6H8 to SO (1 with 70/30, 1 with 60/40, and 4 with 30/70). In our opinion this may be related to the underlying retinal disease, although it is difficult or often impossible to distinguish between problems caused by the tamponade and those associated with the complicated underlying retinal disease. With the mixture in the eye we experienced a percentage of recurrence of PVR of 27.2%, which is within the 6%–35% range reported for SO filled eyes [22, 30, 31]. However, we may assume that an analysis limited to the subgroup of patients who underwent surgery with SO for inferior RD with inferior PVR, and who were unable to maintain a face-down position in the postoperative period, would have shown a recurrence rate of more than 35%.

We experienced a final retinal reattachment rate of 95% after at least one year of followup.

During the follow-up period 1 patient with a F6H8/SO ratio of 70/30 developed a transient IOP increase: this corresponds to 4.5% of the cases, which is lower than the 12%–70% incidence reported in the literature [22]. Five patients (1 of these 5 was operated on twice with F6H8), 3 with ratio of 60/40, 2 with 50/50, and 1 with 30/70, showed low IOP, which remained low throughout followup without any clinical signs of hypotony. In 2 of these patients the F6H8/SO mixture was substituted with SO to prevent development of clinical hypotony. Three patients with a followup of 28 months, 36 months, and 12 months, respectively, still have SO inside the eye. The 5 patients with low IOP represent 22.7% of our cases; this incidence is slightly higher than the 15% hypotony rate reported in complicated RD with conventional tamponades [1]. It has been postulated that hypotony development might be exacerbated by a biological reaction to the heavy tamponade [1]. As mentioned above, although it cannot be affirmed with certainty, hypotony (which was present in 1 of the 5 patients before entering the study), as well as the PVR recurrence rate observed, the transient increase in IOP, the formation of posterior synechiae, and cystoid macular edema occurrence, might be related to the underlying severe retinal disease [32].

We did not clinically observe any obvious emulsification or dispersion in any of the cases. Our results are in substantial agreement with those of Tognetto et al. [22] and Rizzo et al. [28] and might be explained by the absorption of oil bubbles into F6H8 and of F6H8 bubbles into SO.

The F6H8/SO removal time of between 3 and 5 months without signs of emulsification/inflammation further testifies to the intraoperative and postoperative safety of the procedure adopted. Even the patient with compromised general conditions who was followed for 14 months without F6H8/SO removal did not present any signs of inflammation, uveitis, or other problems, except for the formation of a pucker. Moreover, no signs of emulsification/inflammation were observed in the 2 patients who were also subjected to F6H8/SO tamponade in their second operations. Our attention has been focused on the final anatomical results; however, if we consider the critical preoperative conditions of the eyes analyzed in our series, we can also express satisfaction with the final functional results, as 16 out of 22 patients (72%) reached VA better than 20/400.

To our knowledge this sequence of surgical steps renders our technique different from the others and efficacious in avoiding possible side effects related to the intraoperative handling of the substance (e.g., air-heavy tamponade exchange, heavy liquid-heavy tamponade exchange, or heavy tamponade removal), as well as postoperative side effects. Moreover, the outcomes in the eyes of our series treated with different quantitative ratios of F6H8/silicone oil (70/30, 60/40, 50/50, 40/30, and 30/70) are presented: to the best of our knowledge, this has never previously been reported in vivo.

In our opinion F6H8/PDMS is a good alternative in the treatment of inferior RD with inferior PVR, especially for patients, as those of the present series, who are not able to maintain a face-down position in the postoperative period. With a mean followup of 22.63 months, our series, although limited by the relatively small number of patients and by the retrospective nature of the analysis, seems to indicate that the F6H8/SO 1000 cSt mixture is a safe agent for retinal flattening. We can also say that the surgical technique adopted in our case series was found to be very simple and safe.

## Figures and Tables

**Table 1:** Clinical data of patients with inferior retinal detachment and inferior PVR.

Patient	age/sex	Visual acuity at entrance examination	Diagnosis	Associated ocular abnormalities	Previous retinal surgery	Lens status	Surgery with fluoron tamponade	F6H8/SO ratio	Problems with fluoron	Procedure after fluoron use	Fluoron removal (time between use and removal) (months)	Complications	Actual status (VA, retina status)	Followup (months)
1	80/F	CF	RD, PVR	Retinoschisis	None	P	SB-PE-IOLI-Vx-F	70/30	TCT, PS	FR	4	None	CF, A	40
2	34/M	CF	RD, PVR	HM	SB	PP	Vx-F	70/30	TCT	FR	3	Inflammatory reaction 1 y after FR	LP, D	36
3	67/M	CF	RD, PVR	CED	Vx-SO; Vx-SO	AP	SB-Vx-F; Vx-R-F	70/30; 60/40	TCT, PVR	SF-FR	2, 3	None	20/100, A	32
4	68/M	CF	RD, PVR	GL	PE-IOLI-Vx-SO; SB-Vx-SO	PP	Vx-R-F	60/40	TCT	FR	3	None	20/100, A	48
5	67/F	LP	RD, PVR	GL, CED	SB-Vx-SO	PP	Vx-F	60/40	TCT	FR	3	None	CF, A	36
6	80/M	CF	RD, PVR	HM	SB-Vx-G	PP	Vx-F	60/40	TCT, H	FR-SO	3	H	20/200, A, SO not removed	28
7	74/F	MM	RD, PVR	GL	SB	PP	Vx-R-F	60/40	TCT	FR-MP-G	3	None	20/200, A	28
8	78/F	LP	RD, PVR	H	SB-Vx-SO	PP	Vx-F	60/40	TCT, PS, H	FR-SO	4	H	20/200, H, A, SO not removed	36
9	54/M	MM	RD, PVR	GL, HM	T; PE-IOLI-Vx-G	PP	Vx-F; Vx-F	60/40; 50/50	PS, H, TCT, PVR	FR-G	3, 4	H (4 month duration)	20/200, A	18
10	58/M	CF	RD, PVR	None	SB-Vx-SO	PP	Vx-F	50/50	H	FR	3	None	20/100, H, A	16
11	80/F	CF	RD, PVR	HM	None	PP	SB-Vx-MP-F	50/50	F in anterior chamber	FR	3	None	20/200, A	12
12	58/M	CF	RD, PVR	None	SB-Vx-SO	PP	Vx-F	40/60	None	FR	3	None	20/200, A	16
13	70/M	LP	RD, PVR	None	None	PP	SB-Vx-F	40/60	Inferior retinal hemorrhages	FR	5	None	20/100, A	12
14	78/F	CF	RD, PVR	None	SB-Vx-SO	PP	Vx-F	30/70	Superior PVR	FR-R-SO; SOR	3	None	20/100, A	12
15	81/M	20/400	RD, PVR	None	None	PP	SB-Vx-F	30/70	None	FR	3	None	20/70, A	12
16	79/F	LP	RD, PVR	None	None	PP	SB-Vx-F	30/70	Macular PVR, PS	None	—	None	CF, A, F not removed	14
17	84/M	CF	RD, PVR	FB	None	P	PE-SB-Vx-R-F-FBR	30/70	Superior PVR, H	FR, Vx-MP-SO;	1	H	CF, H, A, SO not removed	12
18	76/F	20/400	RD, PVR	None	None	PP	SB-Vx-F	30/70	None	FR	5	CME	20/100, A	12
19	55/F	MM	RD, PVR	None	None	PP	SB-Vx-F	30/70	CME	FR	3	CME (3-month duration)	20/80, A	12
20	80/M	CF	RD, PVR	None	None	PP	SB-Vx-F	30/70	None	FR	3	None	20/100, A	42
21	74/M	CF	RD, PVR	None	None	P	SB-PE-Vx-F	30/70	PS	FR	3	None	20/100, A	12
22	56/F	CF	RD, PVR	HM	PE-IOLI-SB-Vx-G; Vx-SO	PP	Vx-MP-F	30/70	Superior PVR	FR-Vx-MP-SO; SOR	3	None	20/400, A	12

A: attached; AP: aphakia; CED: corneal endothelial dystrophy; CF: count fingers; CME: cystoid macular edema; D: detached; F: F6H8; FB: foreign body; FBR: foreign body removal; FR: F6H8 removal; G: gas; GL: glaucoma; H: hypotony; M: high myopia; IOLI: intraocular lens implantation; LP: light perception; HM: hand motions; MP: membrane peeling; P: phakia; PE: phacoemulsification; PP: pseudophakia; PS: posterior synechiae; PVR: proliferative vitreoretinopathy; R: retinotomy; RD: retinal detachment; SB: scleral buckle; SF: scleral fixation; SO: silicone oil; SOR: silicone oil removal; T: trabeculectomy; TCT: temporary cloudiness of the tamponade; Vx: vitrectomy.
